# Supplementary material for: Unexpected behaviors in molecular transport through size-controlled nanochannels down to the ultra-nanoscale
Source: Nat Commun. 2018 Apr 27;9:1682. doi: 10.1038/s41467-018-04133-8 (PMC5923292; doi:10.1038/s41467-018-04133-8)
Supplement: Supplementary file 1 — Supplementary Information [file 41467_2018_4133_MOESM1_ESM.pdf]

Supplementary Information for

Unexpected behaviors in molecular transport through size-controlled nanochannels down to the ultra-nanoscale

Giacomo Bruno et al.

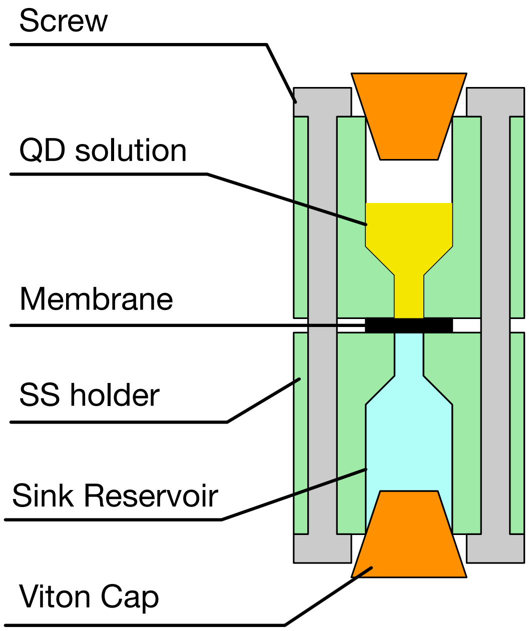

Supplementary Figure 1: Schematics of the quantum dot filtration setup, which employed a stainless steel (SS) holder providing a fluid tight seal on a nanochannel membrane with reservoirs on either side.

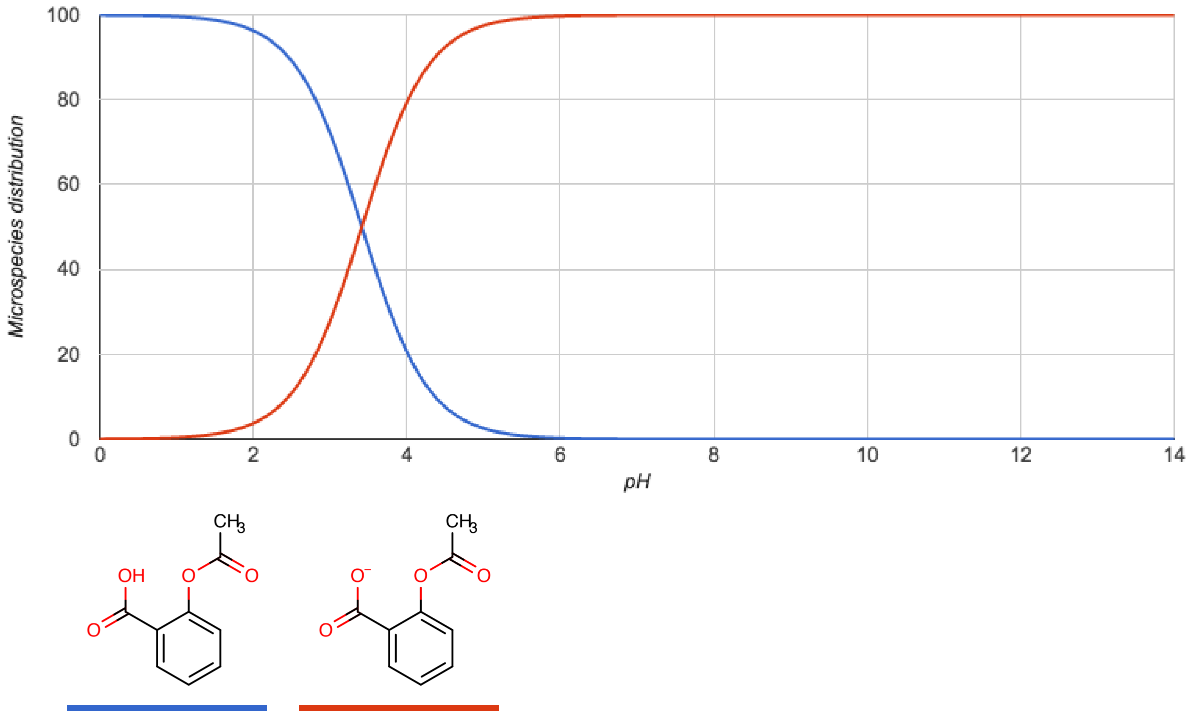

Supplementary Figure 2: **Aspirin**, percentage of microspecies presence in solution at different pH. Data are obtained from chemicalize.org.[12]

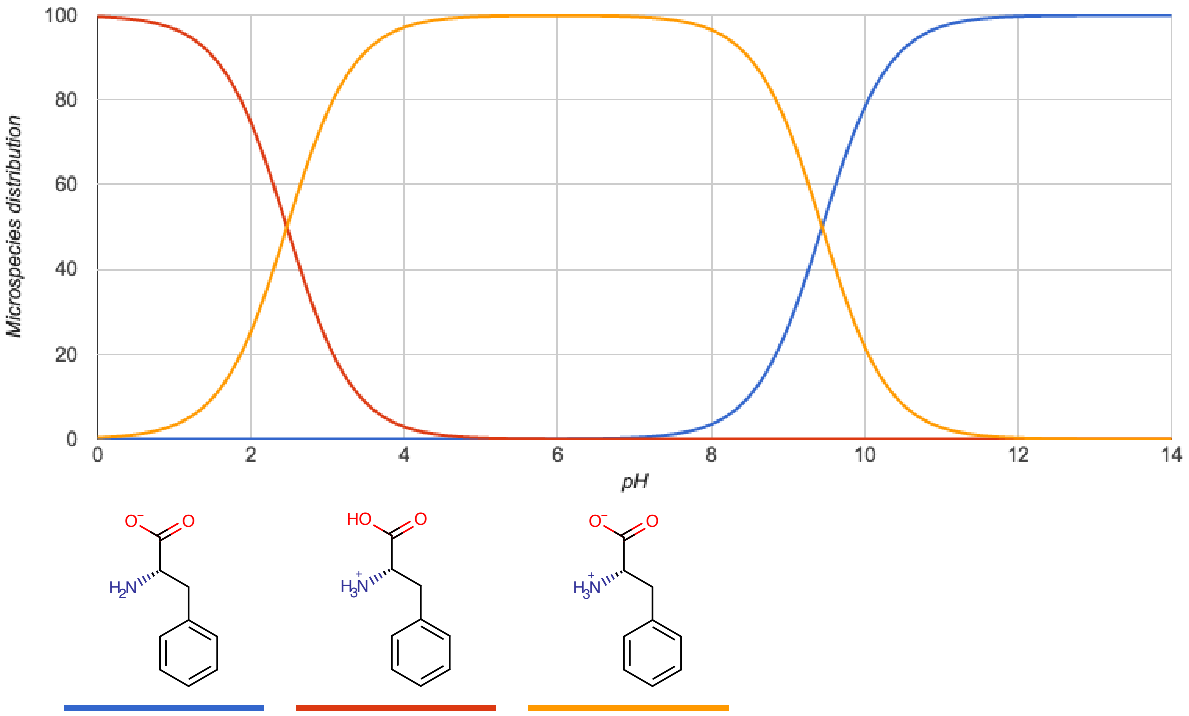

Supplementary Figure 3: **Phenylalanine**, percentage of microspecies presence in solution at different pH. Data are obtained from chemicalize.org.[12]

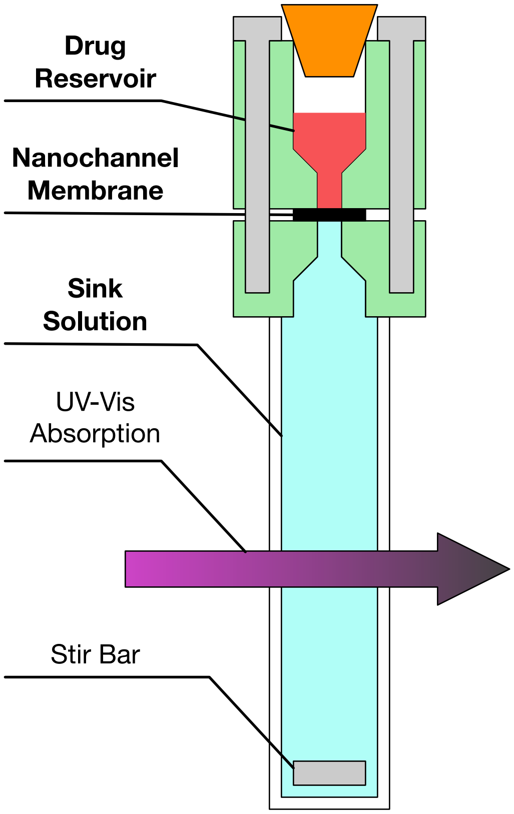

Supplementary Figure 4: Schematic of custom cuvette setup for UV-Vis spectroscopic quantifcation of molecular diffusion.

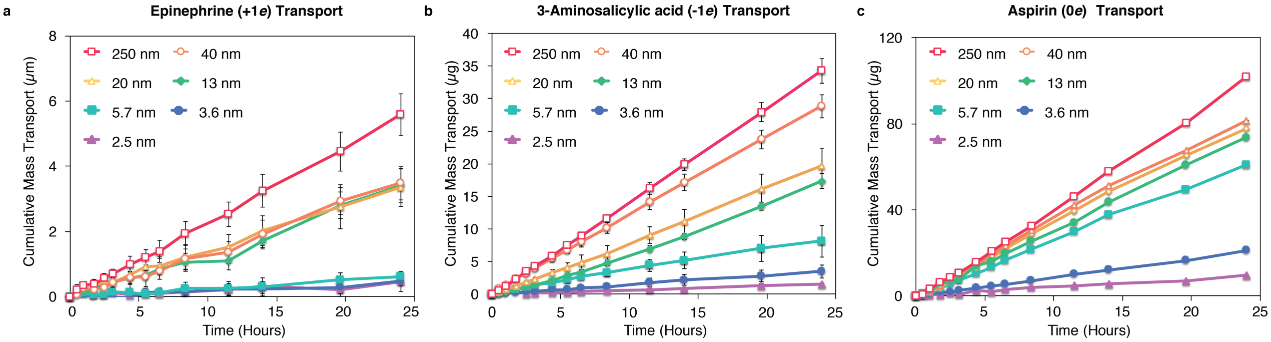

Supplementary Figure 5: Cumulative release profiles of **(a)** epinephrine (positive), **(b)** 3-aminosalicylic acid (negative) and **(c)** aspirin (neutral) through a wide series of nanochannels. Each of the data points denotes the average of three individual replicates, error bars are ± sd.

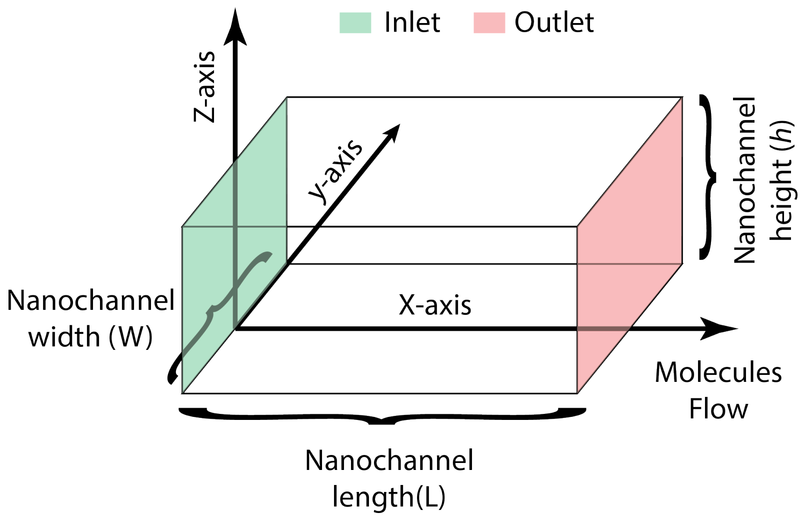

Supplementary Figure 6: Reference axis employed in this model.

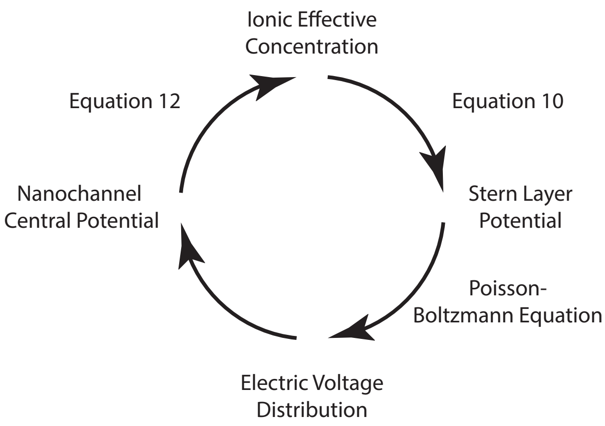

Supplementary Figure 7: Iterative loop to calculate the effective ionic concentration within the nanochannels.

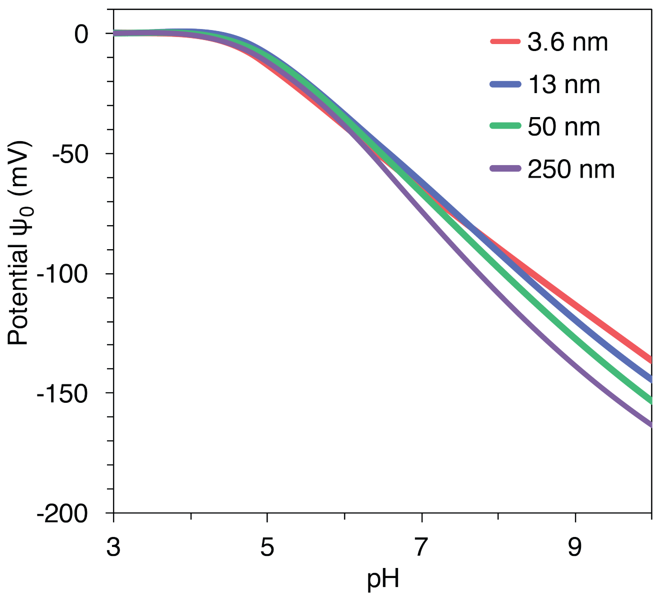

Supplementary Figure 8: Surface potential ψ_0 in different nanochannel size and different solution pH.

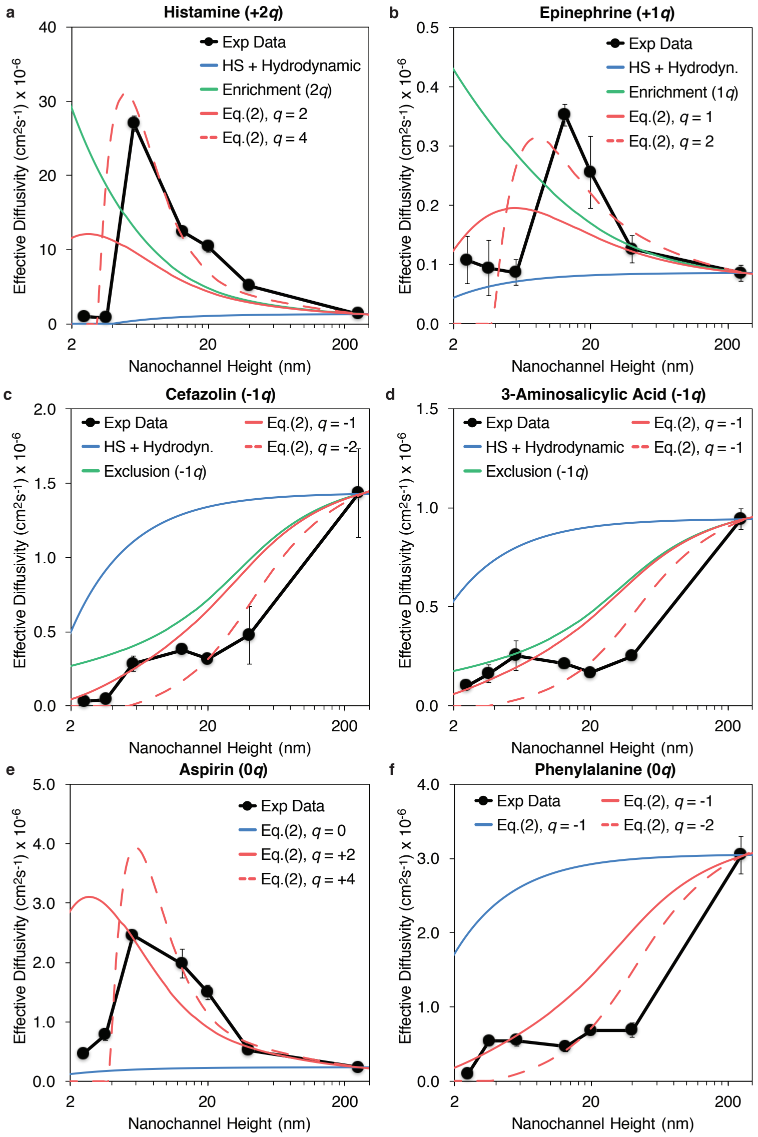

Supplementary Figure 9: Effective diffusivity calculated for each analyte and corresponding theoretical prediction based on Eq. (2) in the main text. Effective diffusivity for positive **(a, b)**, negative **(c, d)** and neutral **(e, f)** molecules. The experimental data [black solid circles] are compared in panels **(a-d)** with Eq. (2) including only hard-sphere (HS) and hydrodynamics effects (solid blue lines), only exclusion or enrichment effects (green solid lines), and the full Eq. (2) (solid red line). The green line is computed from Eq. (2) with rs = 0, corresponding to neglecting HS and hydrodynamic effects. A much better fit can be achieved tuning both the molecule charge, expressed in the parenthesis, and volume [red dash line]. Panels **(e, f)** show diffusivity data for two neutral species. As in the previous panels, blue solid lines are computed from Eq. (2) with q = 0, but a much better fit can be obtained considering the molecules charged. Each of the data points denotes the average of three individual replicates, error bars are $\pm$ sd.

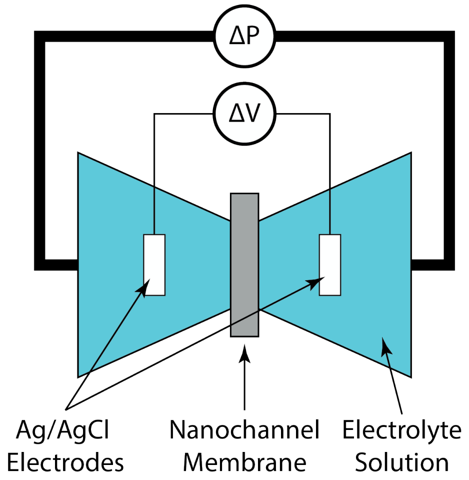

Supplementary Figure 10: Experimental setup for the streaming potential measurement with different applied pressures.

Supplementary Table 1: pH variation inside nanochannels of different sizes with a bulk solution of pH 7 and 50 mM NaCl

nanochannel size (nm)	nanochannel pH
2.5	6.2
3.6	6.26
5.7	6.35
13	6.51
20	6.59
40	6.71
250	6.93

Supplementary Note 1: Geometry and Fabrication Process

The membranes were fabricated using standardized industrial processes at a commercial manufacturer as previously described [8]. Each silicon chip had dimensions of 6 by 6 by 0.750 mm (length, width, and thickness) and enclosed large numbers of monodispersed nanochannels (over 50,000 mm^{-2}). The fabrication protocol began with a Silicon-On-Insulator (SOI) wafer 200 mm in diameter. This wafer had a device layer of 30 μm, a buried oxide layer of approximately 1 μm, and a handle layer of 700 μm. A sacrificial tungsten layer was deposited on the SOI wafer, whose thickness dictated the final nanochannel height. This layer was patterned through photolithography and capped by 20 μm of silicon nitrate. Rectangular outlet microchannels were created vertically with respect to the SOI using a dry etch process through the silicon nitrate layer. The microchannel formation was arrested by reaching the buried oxide layer, exposing the outlets of the nanochannels. The macrochannel inlets, with a dimension of 200 μm by 200 μm, were created by etching the opposite surface of the wafer until reaching the underside of the buried oxide layer. A deep silicon etcher was used to pierce the buried oxide layer and expose the sacrificial layer from beneath, revealing the inlets of the future nanochannels. From this point, hydrogen peroxide etching of the sacrificial material was used to form a contiguous pathway of inlet macrochannels, inlet microchannels, nanochannels, and outlet microchannels. The total number of nanochannels created was consistent across the size range (2.5-250 nm) utilized in all experiments in this manuscript.

The capping dielectric stack, and hence the internal stress associated with these dielectrics, was consistent for nanochannel sizes between 2.5 and 40 nm. The stack thickness was far greater than the nanochannel height, leading to a nearly-identical structure and geometry for all devices. The thickness was changed for the 250 nm channels, as they required a different integration scheme. The stress was high enough to keep the ceiling of the channels tensile in all cases. Following the sacrificial etching process, isopropyl alcohol was substituted for water prior to drying to minimize surface tension and prevent channel collapse. The nanochannel walls were asymmetrically and negatively charged, as the potentials of -28.4 and -18.5 mV were indirectly measured with a zeta potential

analyzer (Delsa Nano C, Beckman Coulter, Inc., CA) for the silicon and silicon nitride surfaces, respectively. The resulting slit-channels, presenting a highly defined and precise geometry with sub-nanometer size tolerances, were parallel to the membrane surface and orthogonal to the inlet and outlet microchannels; a design promoting both high channel density and physical robustness (See Figure 1a) [6].

Supplementary Note 2: Membrane Characterization with SEM and TEM

Figure 1c-i exhibits scanning and transmission electron microscopy (SEM (Nova NanoSEM 230, FEI, OR) and TEM (TEM Analysis, TX)) images to confirm the successful fabrication of nanochannel sizes between 2.5 nm and 250 nm in height. The TEM imaging was performed by the third-party company TEM Analysis. The nanochannel membranes were sectioned prior to imaging with a focused-ion beam system (FEI Strata DB235 SEM/FIB, Hillsboro, OR). The accuracy of the fabrication method is evident in the representative cross-sections with an error less than 10% of the nominal value. This high fidelity employment of well-developed manufacturing techniques allowed significant encroachment into previously unreachable nanoscale resolutions. In addition, this method achieved sufficient nanochannel density to permit significant outflow at a rate easily and reliably detectable by common instrumentation [8], overcoming a primary limitation inherent to similar nanoscale systems [3, 7].

Supplementary Note 3: Membrane Characterization through Gas-Flow Analysis

Silicon membranes were tested by measuring the gas-flow of nitrogen gas (Matheson Tri- Gas, NJ) though the nanochannels. This non-destructive, fast, and highly repeatable method was developed to ensure the fabrication quality of the whole population of silicon channels, addressing a drawback of SEM and TEM imaging inspection (limited to a small portion of one nanochannel). The nitrogen

gas was pressurized at 15 psi by an automated pressure controller (PC3, Alicat Scientific, AZ) and forced to flow though the membrane, which was clamped by a custom-made, stainless steel holder. Leakage within the membrane holder was prevented by a series of silicon o-rings (Apple Rubber, CA). The gas flux was measured by three different mass flow meters (M500SCCM, M10SCCM, M05SCCM, Alicat Scientific, AZ), whose employment depended on the sensitivity required (by dimension of the nanochannels). Over 30 measurements were taken once the pressure and flux reached a steady state for each membrane and averaged. To ensure the quality of the reading and the absence of leakage in the system, a blank test was performed every 15 measurements. This blank test employed a silicon membrane with the same dimension and structure as the membrane previously described, but lacking channels, allowing detection of any gas leakage from non-perfect seals. For calibration and data quality, any measurements reported were associated with blank flow less than the 10% of the smallest reading. Data sampling and system management was performed by a custom-written MATLAB script (The MathWorks Inc., MA), which communicated via serial com ports to the Agilent mass flow meter and pressure controller. This ensured any human variability was minimized and experimental repeatability was maximized.

The nitrogen Q flux through a channel of section A, and length L was modeled as a function of the pressure drop ΔP, as follows:

$$Q = D\frac{A}{L}\frac{\Delta P}{\bar{P}} \tag{1}$$

where $\bar{P}$ ia the average pressure in the channel, and D is the gas diffusion constant, taken to have Knudsen's form:

$$D = \frac{h}{3}\sqrt{\frac{8RT}{\pi M}} \tag{2}$$

where h is the channel height, R the universal gas constant, T the temperature, and M the gas molecular weight. This equation is valid only when the gas is confined within a small space where molecule-to-molecule interactions become insignificant compared to molecule-to-surface interaction. This assumption is only true if the ratio between the gas mean free path and the constraining structure are within a certain range. This comparison is quantified by the Knudsen

number:

$$K_{\mathrm{n}} = \frac{\lambda}{L} \tag{3}$$

where λ is the gas mean free path and L a characteristic length of the system. It is commonly accepted that when $K_{\mathrm{n}} > 0.05$, the system can be described by the Knudsen diffusion equation.

The Knudsen equation was found to reliable predict the gas flow given specified channel geometry and dimensions, as demonstrated by good agreement between the equation and experimental data (see Figure 1j). This test further confirmed the quality of the fabricated membranes.

Supplementary Note 4: Membrane Characterization with Quantum Dot Filtration

CdSe core quantum dots (QDs) (Ocean NanoTech, AR), without substantial surface modification, were solubilized in toluene. QDs were obtained with three different molecular diameters: 2.0, 3.0, and 4.0 nm, which correlated with rated emission peaks at 507, 535, and 584 nm, respectively. The dimensional tolerance was calculated to be ± 0.28 nm, which was determined through interpolating the emission peaks for each QD size. Two identical stainless steel reservoirs, each with a 350 μl capacity, were manufactured to tightly fit either side of the nDS membranes (see Supplementary Figure 1). The membrane was sealed on either side by compression between two Viton O-rings (McMaster-Carr, GA), a material selected for its resistance to the organic solvent. The assembly was held together by two stainless steel screws. The source reservoir was filled with a 200 μl QD solution at a concentration of 100 μg ml^{-1}, while the sink reservoir was filled with $\sim$ 300 μl of pure toluene. Each reservoir was externally sealed with Viton plugs (McMaster-Carr, GA) and JB KWIK epoxy (J-B Weld, TX) to prevent any evaporation. Control experiments were conducted to ensure that none of the materials employed influenced the fluorescent measurement. The experiment was conducted with 2.5 and 3.6 nm nDS membranes and repeated five times (n=5) for each QD size over a 3 day period. Afterwards, the solutions in both chambers were analyzed by a photon-counting spectrofluorometer (PC1, ISS, Champaign, IL) at the rated emission peaks and compared with a negative control where only toluene was added to both reservoirs. The

measurements were normalized to aid comparison.

For the 2.5 nm membrane, a significant difference in permeation between the 2.0 nm and larger QD sizes was evident, while there was no significant difference between the 3.0 and the 4.0 nm QD populations. A similar reduction in permeation was also observed for the 3.6 nm membrane between the 3.0 and 4.0 nm QDs, while the 2.0 and 3.0 nm populations did not differ significantly. Even with a microfabrication tolerance within 10% of the nanochannel's nominal value, these membranes exhibited QD selectivity with a sub-nanometer resolution of $\sim$ 0.5 nm. These results strongly evidenced the nanochannels' precise creation.

Supplementary Note 5: Analytes Employed

Histamine (electric charge $q = +2e$ at pH 4) and epinephrine ($q = +e$ at pH 7) were selected as the model cations for this study. Both are hydrophilic, biogenic amine neurotransmitters. Histamine is composed of an imidazole ring and an amino group connected by two methylene groups, which provides an overall estimated molecular diameter of approximately 0.5 nm. Epinephrine is a cathecholamine consisting of a 1,2-dihydroxybenzene connected by hydroxyl and amine groups to a terminating methyl and has an estimated molecular diameter of approximately 0.6 nm. Acetylsalicylic (0 charge at pH 3) acid and L-phenylalanine (0 charge at pH 7) were employed as model neutral solutes. Acetylsalicylic acid (also known as aspirin) is a commonly employed, non-steroidal anti-inflammatory medication. Its structure consists of a benzene ring connected to carboxylic acid and ester functional groups. L- phenylalanine is an essential amino acid with a phenyl, amino, and carboxyl group sequence. Both molecules have an approximate molecular diameter of 0.6 nm. Cefazolin ($q = -e$ at pH 7) and 3-aminosalicylic acid ($q = -e$ at pH 7) were selected to serve as a model anionic species. Cefazolin is a cephalosporin antibiotic characterized by an elongated conformation and an estimated molecular diameter of approximately 1 nm. 3-aminosalicylic acid is an aminosalicylate employed as an anti-tuberculosis drugs most often with the addition of isoniazid. Its structure consists of a benzene ring connected with amine, hydroxyl, and carboxyl groups and has an approximate molecular diameter of 0.6 nm. Diameters were calculated from estimated molecular volumes [12] and are expected to underestimate the effective hydrodynamic

diameters in aqueous solution. Regarding the two neutral molecule employed in the study, aspirin and phenylalanine, it can be shown from Supplementary Figure 2 and 3, that in the experimental conditions (pH=3 for Aspirin and pH=7 for Phenylalanine), they carry in fact an approximately neutral net charge. Even though Aspirin has a minimal repartition with its negative mircospecie, it's release through the negative nanochannel is negligible due to the high exclusion factor.

Supplementary Note 6: Experimental Setup

Histamine dihydrochloride and acetylsalicylic acid powders were purchased from Sigma- Aldrich (St. Louis, MO). Cefazolin sodium powder was purchased from Baxter (Deerfield, IL). Epinephrine, L-phenylalanine, and 3-aminosalicylic acid were obtained from ThermoFisher Scientific (Waltham, MA). Histamine dihydrochloride (histamine), epinephrine, acetylsalicylic acid (aspirin), L-phenylalanine, cefazolin sodium (cefazolin) and 3-aminosalicylic acid powders were dissolved in 50 mM NaCl in Millipore water at a concentration of 30 mg mL^{-1}, 150 μg mL^{-1}, 1 mg mL^{-1}, 25 mg mL^{-1}, 2 mg mL^{-1}, and 700 μg mL^{-1} respectively. The pH was adjusted using HCl and KOH until the desired pH value was obtained. All membranes were gas tested, piranha cleaned (3:1 H_2SO_4:H_2O_2, v/v), wetted in 2- propanol for several hours, soaked in Millipore water for 3 hours, and immersed in the appropriate solvent (H_2O or 137 mM NaCl in H_2O) for 12 hours prior to diffusion testing. The diffusion testing device assembly shown in Supplementary Figure 4 has been described previously [4]. The wetted membrane was clamped between the sink and source reservoirs. The source reservoir was filled with 150 μl of the solution containing the target molecule. A silicon rubber plug was used to cap the source reservoir. The plug was inserted with a piercing vent needle to remove any trapped air. The needle was removed once the plug was placed. Solution homogeneity was achieved through continuous magnetic stirring with PTFE coated micro-stir bars. Diffusion experiments were performed at controlled RT (23 $\pm$ 0.2 °C) with at least 3 replicates for each channel size: 2.5, 3.6, 5.7, 13, 20, 40 and 250 nm. A custom robotic carousel coupled with an Agilent Cary 50 UV/Vis spectrophotometer, described in a previous publication [5], was used to periodically measure UV absorbance (every 10 min) in the sink reservoir to determine the concentration of histamine (λ_{HI} = 230 nm), epinephrine (λ_{EP} = 279 nm), aspirin (λ_{AP} = 275 nm),

L-phenylalanine ($\lambda_{L-PA} = 257$ nm), cefazolin ($\lambda_{CF} = 280$ nm) and 3-aminosalicylic acid ($\lambda_{3AA} = 315$ nm). Data was normalized with respect to the absorbance at time $t = 0$ and the cumulative mass released was calculated using absorbance vs. concentration standard curves. Details of the experimental procedure, including UV-absorption standard curves, validation of the experimental protocol, and data analysis are described below.

Supplementary Note 7: Cumulative Releases

In order to correlate the UV-Vis absorption of the released molecules with the actual concentration in the sink solution, a standard curve experiment was performed for each molecule used. All the data collected by the automated UV-Vis spectrophotometer were normalized by subtraction of the blank controls. These controls contained the same solution stored in the cuvette sinks to determine an absorbance baseline due to air, water, and the cuvette plastic. The determined time constant value was used to normalize the reading of all diffusion profiles and zero all the cumulative release profiles at the initial time point. The data were then converted from absorption units (arbitrary) to drug concentration leveraging the information previously obtained by the standard curves. To achieve the cumulative released drug over time, the latter data was multiplied by the sink volume (4.450 mL), converting the drug concentration to a finite quantity. It is important to remark that the drugs were proven temporally stable, resulting in the same conversion factors over time. The remaining cumulative releases not published in the main article are plotted in the Supplementary Figure 5.

Supplementary Note 8: Data Fitting

The diffusivity and permeability of the experimental data were found using the following procedure:

- Cumulative release fit with the equation:

$$n_2(t) = \frac{V_1 V_2}{V_1 + V_2} \Delta C_0 \left(1 - e^{-\frac{t}{\tau}}\right) \quad (4)$$

where n_2 is the total mass released at time t into the sink reservoir, V_1 (200 μ l) and V_2 (4.450 mL) the volumes of the source and sink chambers, respectively, ΔC_0 the initial concentration difference while τ is the equilibration time.

- The equilibration time is related to the membrane permeability P_{mem} [cm^{3}s^{-1}] by:

$$\tau = \frac{V_1 V_2}{V_1 + V_2} \frac{1}{P_{\mathrm{mem}}} \tag{5}$$

- Finally, from the permeability, the nanochannel diffusivity D [cm^{2}s^{-1}] is derived. In the simple case of a single channel, permeability and diffusivity are related by: $P_{\mathrm{mem}} = whD/L$, where w, h and L are the width, height and length, respectively, of the channel.

  In our case, the relation between the membrane permeability and the diffusivity in the nanochannels is more complicated, since the permeability contains contributions from the microchannels in the inlet and outlet sections. Taking advantage of the in-series arrangement of the micro- and nanochannels, we can write:

$$P_{\mathrm{mem}} = \left(\frac{L_{\mathrm{i}}}{w_{\mathrm{i}} h_{\mathrm{i}} N_{\mathrm{i}} D_{\mathrm{bulk}}} + \frac{L_{\mathrm{n}}}{w_{\mathrm{n}} h_{\mathrm{n}} N_{\mathrm{n}} D} + \frac{L_{\mathrm{o}}}{w_{\mathrm{o}} h_{\mathrm{o}} N_{\mathrm{o}} D_{\mathrm{bulk}}} \right)^{-1} \tag{6}$$

  where the indices “i”, “n” and “o” refer to “inlet”, “nanochannels” and “outlet”, respectively, N_{i}, N_{n} and N_{o} are the number of micro- or nanochannels in the three respective regions, and D_{bulk} is the bulk diffusivity.

  Using this expression, and assuming that the bulk diffusivity is equal to the value D_{250} measured in the membranes with the largest nanochannels ($h = 250$ nm)

$$P_{250} = D_{250} \left(\frac{L_{\mathrm{i}}}{w_{\mathrm{i}} h_{\mathrm{i}} N_{\mathrm{i}}} + \frac{L_{\mathrm{n}}}{w_{\mathrm{n}} h_{\mathrm{n}} N_{\mathrm{n}}} + \frac{L_{\mathrm{o}}}{w_{\mathrm{o}} h_{\mathrm{o}} N_{\mathrm{o}}} \right)^{-1} \tag{7}$$

  we extract from the measured permeability the nanochannel diffusivity, D. All the relevant lengths are listed in the following table:

	length (L)	width (w)	height (h)
Inlet	15 μm	3 μm	1 μm
Nanochannel	1 μm	3 μm	2.5 - 250 nm
Outlet	1.7 μm	3 μm	1 μm

Supplementary Note 9: The Model Approach

Our approach to modeling the diffusion of charged molecules inside slit-nanochannels of variable heights, containing an NaCl solution at equilibrium between two reservoirs, consists of the following steps:

1. We compute the surface charge density $\sigma_{\rm s}$ on the silica channel surfaces within a site-binding model accounting for the adsorption-desorption of H^+ ions onto SiO^- site, as a function of the electrostatic potential $\psi(z;\nu)$ inside the channel;

2. We acknowledge that the ions in solution have a non-vanishing volume a^3. The electrostatic potential $\psi(z;\nu)$ depends in general on the parameter $\nu = n^0 a^3$, where n^0 is the reference electrolyte concentration in the bulk of the reservoirs; however, for the sake of simplicity we let $\nu = 0$ in most present paper's applications;

3. We compute the electrostatic potential $\psi(z) = \psi(z;0)$ due to point-charge electrolytes inside the slit-channel; for the sake of simplicity, we consider a slit of height h parallel to the z axis, and infinite in the x and y directions, so that the potential only depends on z;

4. We compute from $\psi(z)$ the surface charge density $\sigma_n = \epsilon\epsilon_0 k_{\rm B} T d\psi(z)/dz|_{z=h}$ at the position $z = h$ of the silica-diffuse layer interface;

5. We assume that local equilibrium exists between the charges at the channel surface and in the solution, at the silica-diffuse layer interface, and we set $\sigma_{\rm s} = -\sigma_n$;

6. The computed potential depends in general on two parameters: the value of the potential at the silica-diffuse layer interface, $\psi_{\rm d}$, and the value at the center plane of the channel,

ψ_n. These two parameters are self-consistently computed by iteratively solving $\sigma_\mathrm{s}(\psi_\mathrm{d}, \psi_\mathrm{n}) + \sigma_n(\psi_\mathrm{d}, \psi_\mathrm{n}) = 0$;

7. We then define the effective diffusivity of the diffusing molecules, as the product of a "repartition coefficient" times the bulk diffusivity. The repartition coefficient accounts for the enhancement, respectively decrease, of the transport rate of positive, respectively negative, solutes interacting with the electrostatic potential created by the surface charge and the NaCl solution within the channel. The repartition coefficient also depends on the steric, hard-sphere interactions of the diffusing molecules with the channel walls.

8. All of the following equations are based on the assumption of two infinitely wide planes and they neglect the effect due to the side walls. Additionally, the reference geometry is as illustrated by the Supplementary Figure 6:

Supplementary Note 10: Silica site-binding model

There are several variants of the silica site-binding model, of varying complexity. In general, these models describe how the silica surface charge varies when exposed to a solution of varying pH or ionic concentration. Assuming local equilibrium, the surface charges have to be neutralized by the charges in solution. If we call σ_s the surface charge density, then local equilibrium is guaranteed when

$$\sigma_\mathrm{s} = -\sigma_n \tag{8}$$

where σ_n is the total charge density in the solution. Following Behrens[1] we can define the diffuse layer potential as a function of the surface charge density starting from the Stern layer potential (ψ_0):

$$\psi_0 = \psi_\mathrm{d} - \frac{\sigma_n}{C_\mathrm{S}}. \tag{9}$$

$$\psi_\mathrm{d} = \frac{k_\mathrm{B}T}{e} \ln \frac{\sigma_n}{e\Theta_\mathrm{s} - \sigma_n} + \frac{k_\mathrm{B}T}{e}(\mathrm{pK} - \mathrm{pH}) \ln 10 + \frac{\sigma_n}{C_\mathrm{S}}. \tag{10}$$

where σ_n is the total charge density in the solution, Θ_s is the total concentration of surface sites, $10^{\mathrm{pK}} \equiv K_{-}$ is the equilibrium dissociation constant of the surface sites and C_{S} is the Stern layer's capacity. As we stated in Section SI 1, the channel walls are not symmetric. However, the model we used is extremely simplified, and taking into account a more realistic asymmetry of the surface charge would only add unnecessary complications, without improving the model outcome. We therefore assumed symmetric and equally negatively charged channel walls throughout.

Supplementary Note 11: Poisson-Boltzmann Equation

The electric double layer (EDL) potential in a channel is usually computed from the Poisson-Boltzmann (PB) equation. Our model assumes a symmetrical solvent (NaCl) and identical silica surfaces on both sides of the nanochannel. Recalling the Gouy-Chapman equation, it is possible to obtain the potential variation for a rectangular nanochannel:

$$\frac{d\psi}{dz} = \pm\sqrt{n^0 \frac{8k_{\mathrm{B}}T}{\epsilon\epsilon_0}}\sqrt{\sinh^2\left(\frac{e\psi}{2k_{\mathrm{B}}T}\right) - \sinh^2\left(\frac{e\psi_{\mathrm{n}}}{2k_{\mathrm{B}}T}\right)} \tag{11}$$

Since the surface charge density must balance the charge density in the adjacent solution, it is possible, with the Poisson equation, to relate the surface potential ψ_{d} to the surface charge density σ_n with:

$$\sigma_n = \pm 2\sqrt{n^0 8k_{\mathrm{B}}T\epsilon\epsilon_0}\sqrt{\sinh^2\left(\frac{e\psi_{\mathrm{d}}}{2k_{\mathrm{B}}T}\right) - \sinh^2\left(\frac{e\psi_{\mathrm{n}}}{2k_{\mathrm{B}}T}\right)} \tag{12}$$

where ψ_{d} is the Stern diffuse layer electric potential defined in Supplementary Eq. (10), ψ_{n} is the potential at the nanochannel center-line and n^0 is the ionic concentration of the solution. One may note that the effect of pH variation in the bulk can be accounted for by replacing n^0 with $\widetilde{n}$:

$$\widetilde{n} = 10^3 N_A \left(10^{-pH} + 10^{pH-14}\right) + n^0 \tag{13}$$

Supplementary Eqs. (10) and (12) allow us to completely specify the electrostatic potential $\psi(z)$ inside the channel.

Supplementary Note 12: Effective Diffusivity

The electrostatic potential $\psi(z)$ dictates the distribution of cations and anions inside the nanochannel through the equilibrium relation :

$$n_{\pm}(z) = n^0_{\pm}\text{exp}\left(-\frac{q\psi(z)}{k_{\text{B}}T}\right) \tag{14}$$

where $q = \pm Ze$, and the Z is the ion's valence.

We now assume that when a charged molecule diffuses though the nanochannel it interacts with an external potential $E(z)$, so that its concentration $c(z)$ also follows a Boltzmann distribution:

$$c(z) = c^0 \exp\left(-\frac{E(z)}{k_{\text{B}}T}\right). \tag{15}$$

In particular, we consider $E(z)$ to be the sum of three contributions, $E(z) = E_{\text{HS}}(z)+E_{\text{hyd}}(z)+qZ\psi(z)$. The hard-sphere potential $E_{\text{HS}}(z)$ vanishes everywhere inside the channel, and confines the center of mass of the molecules to be at most at a distance r_{s} from the walls at $z = 0$ and h, where r_{s} is the molecular radius; the term $E_{\text{hyd}} = k_{\text{B}}T \ln[K(2r_{\text{s}}/h, z/h)]$, where $K^{-1}(2r_{\text{s}}/h, z/h)$ is the enhanced drag coefficient (see below) results from hydrodynamic interactions.

In conclusion, the molecular concentration inside the channel is

$$c(z) = \begin{cases} c^0 K^{-1}(2r_{\text{s}}/h, z/h)\text{exp}\left(-\frac{q\psi(z)}{k_{\text{B}}T}\right) & \text{if } \ r_{\text{s}} \leq z \leq h - r_{\text{s}} \\ \\ 0 & \text{elsewhere} \end{cases} \tag{16}$$

where c^0 is an appropriate reference concentration. Note that we are assuming that the diffusing molecules move against a fixed potential and do not modify the charge distribution within the channel. This is clearly not correct, but going beyond this assumption is outside the scope of the paper.

Plecis et al [9] defined a "solute partition coefficient" β, expressed as the ratio between the observed or "effective" permeability P_{eff}, and a "theoretical" permeability P^*:

$$\frac{P_{\text{eff}}}{P^*} = \beta \tag{17}$$

Although Plecis et al. interpret the effective permeability as a consequence of a reduced, or effective, channel height, we follow Deen [2] in defining the diffusivity as the quantity that is modified by the confinement inside the channel, so that:

$$D_{\text{eff}} = \beta D_{250} \tag{18}$$

Still following Deen [2] , we assume that the partition coefficient is proportional to the average of the solute concentration over the channel height, so that:

$$\beta(h) = \frac{1}{h}\int_0^h c(z)/c^0 dz. \tag{19}$$

Supplementary Eq. (16) yields then:

$$\beta(h) = \begin{cases} 0 & \text{if } h \leq r_{\text{s}} \\ \\ \frac{1}{h}\int_{r_{\text{s}}}^{h-r_{\text{s}}} K^{-1}\left(\frac{2r_{\text{s}}}{h}, \frac{z}{h}\right)\exp\left[-\frac{q\psi(z)}{k_{\text{B}}T}\right] dz & \text{if } r_{\text{s}} < h \end{cases} \tag{20}$$

The enhanced drag coefficient has been computed for a rectangular channel using the so called "centerline approximation" [2] which consists in letting $z = h/2$:

$$K^{-1}\left(\frac{2r_{\text{s}}}{h}, \frac{z}{h}\right) \approx K^{-1}\left(\frac{2r_{\text{s}}}{h}, \frac{1}{2}\right) \approx \tag{21}$$

$$\approx \left[1 - 1.004\left(\frac{2r_{\text{s}}}{h}\right) + 0.418\left(\frac{2r_{\text{s}}}{h}\right)^3 + 0.21\left(\frac{2r_{\text{s}}}{h}\right)^4 - 0.169\left(\frac{2r_{\text{s}}}{h}\right)^5 + O\left(\frac{2r_{\text{s}}}{h}\right)^6\right] \tag{22}$$

Finally:

$$\beta(h) = \begin{cases} 0 & \text{if } h \le r_{\mathrm{s}} \\ \\ \frac{1}{h} K^{-1}\left(2\frac{2r_{\mathrm{s}}}{h}, \frac{1}{2}\right) \int_{r_{\mathrm{s}}}^{h-r_{\mathrm{s}}} \exp\left[-\frac{q\psi(z)}{k_{\mathrm{B}}T}\right] dz & \text{if } r_{\mathrm{s}} < h \end{cases} \quad (23)$$

In the case of a neutral solute ($q = 0$) the partition coefficient simplifies to [10, 11, 2].

$$\beta_{\mathrm{neutral}} = \begin{cases} 0 & \text{if } h \le r_{\mathrm{s}} \\ \\ K^{-1}\left(\frac{2r_{\mathrm{s}}}{h}, \frac{1}{2}\right)(h - 2r_{\mathrm{s}})/h & \text{if } r_{\mathrm{s}} < h \end{cases} \quad (24)$$

A coefficient $\beta(h) > 1$ for a given class of molecules implies that their concentration inside the nanochannel is larger than the reference one: this situation is called *enrichment*. On the contrary, $\beta(h) < 1$ for a class of ions implies that those ions are prevented to enter the channel, leading to a lower concentration than in the reference state; this is called *exclusion*.

Supplementary Note 13: Model Implementation

Calculations are based on the self-consistent numerical solution of the Poisson-Boltzmann equation, coupled to the site-binding model of the silica surface charge. Knowing the electrostatic potential within the nanochannel requires knowledge of its value at the Stern layer, ψ_{d}, and on the center plane of the channel, ψ_{n}. The first derivative of the electrostatic potential, computed from Supplementary Eq.(11) yields the Stern layer potential with the use of Supplementary Eq. (9); the effective surface charge within the nanochannel can then be calculated from Supplementary Eq. (12). This quantity must be equated to the surface density of exposed charges on the silica surfaces, which in turn depends on the value of the Stern layer potential itself. An iterative loop was employed starting from a uniform distribution of ions in the nanochannels identical to the bulk value. A stable solution is always possible as the cycle employs a negative feedback loop: the increase in ionic concentration produces a decrease in the Stern potential and vice versa (as

represented in Supplementary Figure 7).

This iterative method, therefore, produce results that are unique for both the nanochannel size and bulk solution parameters such as ionic concentration and pH. Supplementary Figure 8 illustrates the variation of surface potential inside the nanochannel for a solution of 50 mM NaCl and different pH.

Once convergence of the ionic concentration and of the potential is obtained, it is possible to calculate the partition coefficient (Supplementary equation 24) and the effective diffusivity (Supplementary equation 18) for a given solute.

Supplementary Note 14: Nanochannel pH

As described in the previous section, the model iteratively calculates the concentration of ions and, consequently, the concentration of hydronium (H^+) molecules. We were able to observe that the enrichment factor cause an increase H^+ concentration and, therefore, a reduction of pH. This phenomenon, in agreement with Plecis [9], is predominant in small channels or at low ionic strength. With this model, we predicted for our smallest nanochannels a decrease in pH to 6.2 from its bulk value 7 (see Supplementary Table 1). The molecules investigated here were chosen in such a way that their physical and chemical properties would not change within such a variation in pH.

Supplementary Note 15: Modeled Diffusivity

This model was compared to experimental diffusivities in Fig. 3 of the main article. Here, we vary the parameters Z and r_s in such a way to obtain the best possible fit to the measurements, irrespective of the physical interpretation of the fitting values. Supplementary Figure 9 shows the best fit as a dashed red line. For positive molecules (Supplementary Figure 9a and 9b) the best fit is found by multiplying the valence by 2 and the molecular diameter by 4. In the case of negative molecules (Supplementary Figure 9c and 9d) a similar variation of Z and r_s leads to a lowering of the computed diffusivity, with a better quantitative "fit", but still a shape that does match

the measurements. Similar considerations apply to neutral molecules, for which this "fit" is even harder to justify.

Supplementary Note 16: Streaming Potential

Measuring the streaming potential allows one to estimate the ζ potential at the silica surfaces of the nanochannels. Applying a mechanical pressure between the nanochannel inlet and outlet forces the molecules to pass through the membrane. The silica surface charges create within the nanochannel an electric field, favoring the flow of counter-ions over that of the co-ions, producing a net ionic current. By doing so, the streaming potential starts to build up between the two floating Ag/AgCl electrodes (see Supplementary Figure 10). At steady state, the potential gradient (U_{str}) is directly proportional to the ζ potential (ζ) and to the pressure gradient (ΔP) as follows:

$$U_{str} = \frac{\epsilon_{\mathrm{r}}\epsilon_0\zeta}{\eta K_L}\Delta P \tag{25}$$

where ϵ_{r} and ϵ_0 are respectively the relative and electrical permittivity, η the dynamic viscosity, and K_L the specific conductivity of the bulk solution. Employing the Smoluchowski theory as a first approximation, since the thin double layer assumption is not always valid, we can assume $\psi\left(\kappa^{-1}\right) \approx \zeta$ where κ^{-1} is the Debye length. Finally, thanks to the Poisson-Boltzmann equation, the corresponding surface electric potential (ψ_0) can be calculated and compared with the one computed from the model.

Supplementary References

[1] S. H. Behrens and D. G. Grier. The charge of glass and silica surfaces. *J. Chem. Phys.*, 115(14):6716–6721, 2001.

[2] W. Deen. Hindered transport of large molecules in liquid-filled pores. *AIChE Journal*, 33(9):1409–1425, 1987.

[3] S. Ferrati, D. Fine, J. You, E. De Rosa, L. Hudson, E. Zabre, S. Hosali, L. Zhang, C. Hickman, S. S. Bansal, et al. Leveraging nanochannels for universal, zero-order drug delivery in vivo. *J. Control. Release*, 172(3):1011–1019, 2013.

[4] C. V. Fletcher, K. Staskus, S. W. Wietgrefe, M. Rothenberger, C. Reilly, J. G. Chipman, G. J. Beilman, A. Khoruts, A. Thorkelson, T. E. Schmidt, et al. Persistent hiv-1 replication is associated with lower antiretroviral drug concentrations in lymphatic tissues. *Proc. Natl. Acad. Sci.*, 111(6):2307–2312, 2014.

[5] T. Geninatti, E. Small, and A. Grattoni. Robotic uv-vis apparatus for long-term characterization of drug release from nanochannels. *Meas. Sci. Technol.*, 25(2):027003, 2014.

[6] D. S. Gianola and J. Shin. Nanomechanics: Full recovery takes time. *Nat. Nanotechnol.*, 10(8):659–660, 2015.

[7] A. Grattoni, J. Gill, E. Zabre, D. Fine, F. Hussain, and M. Ferrari. Device for rapid and agile measurement of diffusivity in micro-and nanochannels. *Anal. Chem.*, 83(8):3096–3103, 2011.

[8] A. Grattoni, H. Shen, D. Fine, A. Ziemys, J. S. Gill, L. Hudson, S. Hosali, R. Goodall, X. Liu, and M. Ferrari. Nanochannel technology for constant delivery of chemotherapeutics: beyond metronomic administration. *Pharm Res*, 28(2):292–300, 2011.

[9] A. Plecis, R. B. Schoch, and P. Renaud. Ionic transport phenomena in nanofluidics: experimental and theoretical study of the exclusion-enrichment effect on a chip. *Nano Lett.*, 5(6):1147–1155, 2005.

[10] F. G. Smith and W. M. Deen. Electrostatic double-layer interactions for spherical colloids in cylindrical pores. *J. Colloid Interface Sci.*, 78(2):444–465, 1980.

[11] F. G. Smith and W. M. Deen. Electrostatic effects on the partitioning of spherical colloids between dilute bulk solution and cylindrical pores. *J. Colloid Interface Sci.*, 91(2):571–590, 1983.

[12] M. Swain. Chemicalize. org, 2012.
